# Food environments and the COVID-19 pandemic in Brazil: analysis of changes observed in 2020

**DOI:** 10.1017/S1368980021003542

**Published:** 2022-01

**Authors:** Larissa Loures Mendes, Daniela Silva Canella, Melissa Luciana de Araújo, Mariana Zogbi Jardim, Letícia de Oliveira Cardoso, Milene Cristine Pessoa

**Affiliations:** 1 Nutrition Department, Federal University of Minas Gerais, Avenue Alfredo Balena, 190, Room 314, Santa Efigênia, Belo Horizonte, MG 30130-100, Brazil; 2 Institute of Nutrition, Rio de Janeiro State University, Rio de Janeiro, RJ, Brazil; 3 Program in Health and Nutrition, School of Nutrition, Federal University of Ouro Preto, Morro do Cruzeiro, Ouro Preto, MG, Brazil; 4 Program in Child and Adolescent Health, Federal University of Minas Gerais, Belo Horizonte, MG, Brazil; 5 Epidemiology Department, National School of Public Health, Oswaldo Cruz Foundation, Rio de Janeiro, RJ, Brazil

**Keywords:** Food environments, COVID-19, Nutrition, Public health

## Abstract

Evidence of changes caused by the COVID-19 pandemic in food security and nutrition conditions, as well as in different food environments, has called researchers’ attention to substantial changes taking place in individuals’ dietary habits. The aim of this study is to present and address changes that have already happened in food environments, during the first COVID-19 pandemic year, in a middle-income country. Multiple changes were observed and had direct impact on the population, among them, worsened health and nutrition indicators and advanced dietary inequalities, as well as on its food profile in different life cycles, if one takes into consideration aspects such as food availability, financial access and dietary quality.

There is an evidence that the global pandemic triggered by the emergence and spread of the new human coronavirus (SARS-CoV-2), which was declared by the WHO in March 2020^(1)^, has had effects on food systems worldwide. Food environments in most countries have rapidly experienced changes in their external dimensions, such as food availability, prices, suppliers, as well as in personal dimensions, such as geographic accessibility, affordability and convenience. Rapid changes in food environments can influence consumers’ eating habits, as well as worsen national health and nutrition indicators^(2–4)^.

One year after the beginning of the COVID-19 pandemic, several uncertainties about changes in its features remain in different food environments and contribute to the advancement of food and nutrition insecurity^(5–8)^, as well as to worsen health, income, employment and education conditions^(9–13)^. The aim of this study is to present and address changes that have already taken place in food environments during the first COVID-19 pandemic year in a middle-income country.

## Food environments and their modifications

At the beginning of the COVID-19 pandemic in Brazil and in several countries worldwide, the fear of food shortage has led part of the population to purchase great amounts of processed and ultra-processed food due to its durability, practicality and easy access in different physical and online food environments^(3,14)^. Moreover, consumers’ perception about the risks they are exposed to and the impact of this perception on their intention and decision to purchase food should also be taken into consideration. Thus, the perception of risk associated with food supply at the beginning of COVID-19 pandemic has influenced individuals’ eating behaviour^(3)^ and led them to use delivery services rather than going to the physical store.

The worsening of the COVID-19 pandemic in several Brazilian cities has led to the need of taking harder measures about social distancing, as well as to security determinations focussed on reducing human circulation and the consequent spread of the virus. These measures comprised closing public food procurement sites such as municipal markets and open fairs, as well as restricting physical access to establishments selling food for immediate consumption such as restaurants and snack bars^(2,15)^. In addition, many traders have changed the selling pattern of their establishments in order to meet community demands and to reduce the impact of the crisis^(16)^. These measures have contributed to change the physical retail food environment^(17)^ through the migration of many establishments, mainly those that sell food for immediate consumption, to the virtual environment^(2)^.

These sudden changes have significantly changed consumers’ profile since they started purchasing food through several applications and websites of the most varied food-selling establishment categories. Digital food environment has undergone significant increase in takeout/away and delivery services during the pandemic, mainly because Brazilian regulations have ordered many mass catering establishments to be closed, whereas many others had to migrate to the virtual environment^(2)^.

Unhealthy—ultra-processed food types, such as hamburgers, pizzas, sweets in general, sugary drinks, among others, ‘are more available’ in these food purchase convenience channels^(18,19)^. The access of different consumers to the digital environment, mainly due to issues such as physical distance between the residences and the establishments, convenient access to food and beverages^(19)^ and overload of household activities, may contribute to the development of unhealthy eating habits in the mid and long term^(20,21)^.

The digital food environment—which has been rapidly expanding in Brazil^(19)^—presents similar profile to all other digital categories when it comes to the influence of advertising on individuals’ food choices. This factor implies food promotion and advertising in the most different media and it may lead to unhealthy food choices^(7,22)^.

Consumers have been facing issues in the food environment such as worsened food affordability^(23)^. Data have shown increased price of basic food items such as rice, beans and coffee powder, since the beginning of the pandemic in Brazil. Calculations made by the Inter-Union Department of Statistics and Socioeconomic Studies (DIEESE), based on data from the National Basic Food Basket Survey, have shown an increase in all 17 surveyed capitals that have recorded the highest annual increase in food prices in 2020^(23)^.

In addition, consumers’ loss of purchasing power had significant impact on family farming, since farmers depend on local supply and on decentralised food marketing systems^(24)^ to generate income. Furthermore, a study about socioeconomic conditions in Brazil has pointed out remarkably negative impacts of the COVID-19 pandemic on family income^(25)^, which can substantially lead to deleterious effects on food purchasing, mainly when it comes to the quantity and quality of purchased products. Unexpectedly some web surveys results are not showing a worsening diet quality during the pandemic period. The Nutrinet Brazil survey, which started in January 2020, back in May, 2020, showed slight increase in the consumption of healthy eating markers and stability in the consumption of unhealthy eating markers in most sociodemographic strata during the COVID-19 pandemic, except for the Northern and Northeastern macroregions in Brazil, which showed trend of increased ultra-processed food consumption^(26)^. However, results should be interpreted with caution, since it is a cohort study conducted with non-probabilistic sample and with reduced absolute number of participants in some macroregions and income strata under greater vulnerability condition^(26)^.

Most changes in food environments resulting from the pandemic may be contributing to modify food consumption among Brazilians. The Brazilian survey ConVid, conducted between April and May 2020, during the social distancing period, pointed out higher alcoholic beverage intake, decreased frequency of healthy food intake and increased frequency of unhealthy food intake due to the COVID-19 pandemic, except among the elderly population^(27)^.

Accordingly, a cross-sectional study was conducted with 820 adolescents in the age group 10–19 years, from different countries, including Brazil, during the pandemic, from April to May 2020. It was based on an online questionnaire distributed by social media such as *WhatsApp* and *Twitter*, and showed changes in food intake. On the one hand, there was increased vegetable intake, mainly in Brazil. On the other hand, the mean fried food and sweet intake have significantly increased during the forced confinement imposed on participants due to the COVID-19 pandemic^(28)^.

With respect to the school food environment, it is known that physical distancing measures have led to school closure and had negative impact on students’ education and nutritional status. When it comes to public schools, the National School Food Program (*Programa Nacional de Alimentação Escolar*) accounts for ensuring adequate food for Brazilian schoolchildren during school days^(29)^. However, this process was initially interrupted by the pandemic; besides, the inefficient and delayed federal guidance by the National Education System regarding National School Food Program has generated multiple and discrepant actions by state and local authorities^(30)^. Thus, on 7 April 2020, the distribution of foodstuffs purchased with federal funds to schoolchildren’s parents or legal guardians was officially authorised countrywide^(31,32)^.

With respect to school feeding strategies adopted by state and local governments, some places used their own resources to deliver non-family farming food baskets while others performed direct cash transfer through food card/value,^(29,30)^ and some, to a lesser extent, delivered food baskets purchased from small family farmers^(30)^.

It is noteworthy that distributing food baskets without properly planning the nutritional quality of the provided food can undermine the work of Food and Nutrition Education (*Educação Alimentar e Nutricional*), which has been carried out by National School Food Program over the years. The aim of Food and Nutrition Education, which is in line with the dietary guidelines for the Brazilian population^(33)^, is to promote the intake of fresh and healthy foods such as fruits, vegetables and legumes, including food items belonging to local cultures and farmers. Its interruption can damage the adequate and healthy diet of schoolchildren^(34)^.

Studies conducted before the COVID-19 pandemic have shown that private schools presented a more obesogenic food environment than public schools^(35)^. Face-to-face classes in private schools were quickly replaced by online classes; however, the same did not happen in public schools. Also, the excessive use of electronic devices and lack of contact with peers can influence sleep patterns, change eating habits, as well as trigger stress and anxiety behaviours^(36)^. Cafeteria owners who sell food in private schools have seen their economic activity suddenly paralysed and suffered great economic impact due to the COVID-19 pandemic. Consequently, changes in ownership, in places where food is sold and in the type of food sold are expected when presential activities return. Thus, it is of paramount importance to think about strategies to reverse, rather than to worsen, the obesogenic food environment in private schools when the classroom activities return.

## Final considerations

The conditions generated by the pandemic in recent months, mainly when lockdown and physical distancing were adopted as strategies to stop disease transmission, have reduced individuals’ access to healthy food and contributed to increased social and dietary inequalities, as well as to disparity in health-related behaviours. This scenario has considerably influenced the nutritional condition of the population, such as nutritional status, hunger and food and nutrition insecurity^(29,37)^.

Besides the web surveys have been showing some healthy diet trends at different times in 2020, the COVID-19 pandemic is expected to increase dietary inequalities expressed in different food environments, at their different dimensions. It is essential and demanding to address different food environments, as well as their reflex on human health, mainly in the scope of regulatory policies focussed on promoting the availability of, and financial access to, food.

The retail food environment is undergoing several changes associated with the availability of and financial accessibility to food during the current COVID-19 pandemic. These changes have a direct impact on populations’ eating profile, which, in its turn, affects their eating habits and triggers nutritional issues.

Facing this scenario, some actions could be taken in the short, medium or long term. In the short term, confronting COVID-19 requires that school feeding programs continue to be designed for school children’s families, promoting support for small farmers and family farming^(38)^. Actions such as encouraging cooking skills, growing one’s own food and reducing food waste are also necessary^(39)^. In the medium and long term, actions to address nutritional issues such as food insecurity and obesity by strengthening intersectoral health actions are important and include compliance with health protocols to ensure the supply, access, promotion, purchase and consumption of healthy foods^(7)^. Strengthening actions for food labelling and urban planning that encourages physical activity are also relevant strategies^(39)^.

Finally, it is worth emphasising that the catastrophic situation observed in Brazil during the COVID-19 pandemic and the political inertia towards food and nutritional security actions may result in the potential worsening of health and nutrition indicators^(10)^. In addition, Brazil will likely remain on the hunger map in the coming years, as well as face the risk of increasing obesity prevalence in all life cycles.
